# Epidemiology of potential drug-drug interactions in elderly population admitted to critical care units of Peshawar, Pakistan

**DOI:** 10.1186/s40360-018-0276-4

**Published:** 2018-12-10

**Authors:** Faisal Shakeel, Muhammad Aamir, Ahmad Farooq Khan, Tayyiba Nader Khan, Samiullah Khan

**Affiliations:** 1 Department of Pharmacy, Sarhad University of Science and IT, Peshawar, Pakistan; 2 University of Vermont Medical Centre, Burlington, VT USA; 3 Punjabi Community Health Services, Calgary, Alberta Canada; 4 Department of Pharmacy, University of Malakand, Lower Dir District, Pakistan

**Keywords:** Geriatrics, Potential drug-drug interactions, Epidemiology

## Abstract

**Background:**

Aging population, is a reality in many countries because of improvement in the health care, patient safety and other supplemental factors. Pharmacotherapy in this population must be evaluated due to their higher susceptibility to adverse drug outcomes, like potential drug-drug interactions (PDDIs). Research in this regard is limited particularly in developing countries. The aim of the study was to evaluate the prevalence and associated factors in this population.

**Methods:**

The multicentered study evaluated the prevalence of potential drug-drug interactions and associated factors in elderly population at critical care units in Peshawar, Pakistan. Potential drug-drug interactions were evaluated using Micromedex DrugReax, while statistical analysis was performed using SPSS.

**Results:**

A total of 70.17% elderly patients were observed to have at least one PDDI. A significant association was observed between presence of PDDIs and number of prescribed drugs, duration of stay and age (*p* < 0.05). A total of 3019 PDDIs were observed, attributing to 225 drug pairs. Prevalent PDDIs were of moderate severity, good documentation and pharmacodynamic in nature. One-way ANOVA revealed a significant difference in the means of PDDIs between Northwest general hospital and the rest of the hospitals. Moreover, there was a significant difference in the means of PDDIs of CCU and SU with rest of the units.

**Conclusion:**

The prevalence of PDDIs was observed to be high in elderly population which can be managed by avoiding or managing a limited number of drug combinations. Such studies are necessary to evaluate the risks of these PDDIs in a population which is already physiologically compromised.

**Electronic supplementary material:**

The online version of this article (10.1186/s40360-018-0276-4) contains supplementary material, which is available to authorized users.

## Background

An outcome of interrelated developmental achievements is an ageing population. Improvement in healthcare is a major factor for increasing life expectancy, along with supplemental factors like improved nutrition, education, income and sanitation [1]. Patient safety has gained much attention in recent years by health care providers, further incrementing the age of the population. Pharmacotherapy has aided in improving health of the patients, however, it has also led to a rise in adverse drug events. One such adverse event is drug-drug interactions [2].

Elderly population, known as geriatrics, face many health issues due to the natural process of ageing, beyond the control of humans. Treating multi-morbidities in geriatrics with drugs is complex and leads to the expression of adverse drug events. This along with the natural functional impairment tends to impart harm rather than benefit in geriatrics [3, 4].

Drug-drug interaction is the modification, increase or decrease in the effects of drugs when simultaneously administered with another drug. This leads to severe adverse effects which are totally preventable in most cases if suitably managed [5]. Geriatric population is at a higher risk to these interactions due to the natural functional impairment, and identification of the drug-drug interactions in this population becomes imperative [6, 7].

In Pakistan, monitoring of pharmacotherapy in geriatrics in neglected. Moreover, the prevalence of drug-drug interactions is also not known. Thus, understanding the mechanisms and factors involved in these potential drug-drug interactions (PDDIs) is important to aid the prevention of adverse effects of the interacting drug pairs.

## Methods

The multicentered cross sectional study was conducted at the critical care units of four tertiary care hospitals in Peshawar, Pakistan; Lady Reading Hospital (LRH), Khyber Teaching Hospital (KTH), Hayatabad Medical Complex (HMC) and Northwest General Hospital and Research Center (NWGH & RC). The former 3 hospitals are government run, while the latter is a private hospital. The critical care units included were Medical Intensive Care Unit (MICU), Surgical Intensive Care Unit (SICU), Cardiac Care Unit (CCU), and Stroke Unit (SU). Patients from the Northwestern region of Pakistan and Afghanistan avail the medical facilities in these hospitals. Patients who fulfilled the inclusion criteria were randomly selected from these critical units. Inclusion criteria was set as patients of age of 60 years or above, prescribed 2 or more drugs, and admitted to the critical care unit for more than 24 h. Data of 2960 patients was collected over the period of 1 year (Dec 2013 – Dec 2014) of which 1044 met the inclusion criteria.

Prior to collection of data, ethical approvals from the respective hospitals were obtained beforehand vide letter number 8075–79/HMC, 488/pharm (KTH), 010 (LRH), and NWGH/Research/01. A predesigned proforma was used to collect the patient demographic data (age, gender, hospital, unit, date of admission and discharge) and treatment profile (diagnosis, drugs administered, dose and frequency, duration of drug administration); patient identification and other personal data were not disclosed. ICH guidelines for good clinical practice were followed [8]. Written informed consent was not necessary because no personal patient data has been included in the manuscript and data was collected from the medication charts of the patients, for which the hospital ethical committee provided approval.

Evaluation of drug-drug interactions were carried out through Micromedex DrugReax [9] which provides details on the severity, documentation, onset and mechanism of the PDDIs. Severity is classified by Micromedex as major, moderate and minor, while documentation is classified as excellent, good and fair. Micromedex also elaborates the mechanism of the interacting drug pairs. Drugs administered simultaneously during treatment were evaluated for PDDIs.

Statistical analysis was performed using IBM SPSS Statistics for Windows, Version 20 (Armonk, NY: IBM Corp.) [10]. Various statistical tools were used for analysis of descriptive data, along with logistic regression to evaluate the association of PDDIs with predictive factors. One-way ANOVA was also employed to observe the difference in means of PDDIs among the four hospitals.

## Results

Of the total 1044 patients included in the study, 877 (84%) aged ≤75 years while 167 (16%) patients aged > 75 years. Male patients were predominant (60.3%) as compared to female patients (39.7%). The mean stay of the patients in critical care units was 4.56 ± (3.12) days, while the mean number of prescribed drugs was 5.99 ± (1.88) drugs. CCU saw the highest flow of inpatients as compared to other units. Similarly, patients with a primary diagnosis of myocardial infarction were predominant, as shown in Table 1.

**Table 1:** Demographics and general characteristics of study population (*N* = 1044)

Variables	Mean ± SD	Frequency (%)	Range
*Gender*	–		–
Male	–	630 (60.3%)	–
Female	–	414 (39.7%)	–
*Age (years)*	68.53 (± 7.81)	–	60–100
*Drugs prescribed per patient*	5.99 ± (1.88)	–	2–13
*Stay in ICU (days)*	4.56 ± (3.12)	–	1–38
*Critical Unit*	–	1044 (100%)	–
Surgical ICU	–	151 (14.5%)	–
Medical ICU	–	264 (25.3%)	–
Cardiac ICU	–	499 (47.7%)	–
Stroke Unit	–	130 (12.5%)	–
*Hospital*	–	1044 (100%)	–
NWGH	–	420 (40.2%)	–
LRH	–	174 (16.7%)	–
KTH	–	223 (21.4%)	–
HMC	–	227 (21.7%)	–
*Diagnosis*	–	1044 (100%)	–
Myocardial Infarction	–	261 (25.00%)	–
Cerebrovascular Accident	–	96 (9.20%)	–
Acute Coronary Syndrome	–	82 (7.85%)	–
Heart Failure	–	59 (5.65%)	–
Chronic Obstructive Pulmonary Disorder	–	34 (3.26%)	–
Miscellaneous	–	512 (49.04%)	–

*ICU* Intensive care unit, *NWGH* Northwest General Hospital, *LRH* Lady Reading Hospital, *KTH* Khyber Teaching Hospital, *HMC* Hayatabad Medical Complex, *SD* Standard Deviation

Prevalence of potential drug-drug interactions (PDDIs) was 71.07% of all the patients. A total of 137 (13.1%) patients were observed to have at least one PDDI, 110 (10.5%) had 5 PDDIs, while 2 (0.2%) had 19 PDDIs. PDDIs were most prevalent in patients with myocardial infarction, as shown in Table 2.

**Table 2:** Characteristics of potential drug-drug interactions

Variables	No. of Patients (%)
*Potential drug-drug interactions*	
Present	742 (71.07%)
Absent	302 (28.93%)
*Most severe PDDI seen in each patient*	
Contraindicated	6 (0.81%)
Major	618 (83.29%)
Moderate	113 (15.23%)
Minor	5 (0.67%)
*Diseases with highest prevalence of PDDIs*	
Myocardial Infarction	257 (34.64%)
Cerebrovascular Accident	82 (11.05%)
Acute Coronary Syndrome	79 (10.65%)
Heart Failure	44 (5.93%)

A total of 3019 PDDIs were observed in 1044 patients, of which 1398 (46.3%) were of major severity, 1533 (50.8%) were of moderate severity, 82 (2.7%) were of minor severity while 6 (0.2%) PDDIs were contraindicated. In terms of documentation, 372 (12.3%) PDDIs were of excellent, 1485 (49.2%) PDDIs were of good, and 1162 (38.5%) PDDIs were of fair documentation. The onset of 1758 (58.2%) PDDIs was unknown, while 529 (17.5%) and 732 (24.2%) PDDIs were of rapid and delayed onset respectively. Pharmacodynamic nature PDDIs (66.5%) were common, while synergistic mechanism (44%) was predominantly involved in the PDDIs.

Multivariate logistic regression was applied to associate multiple predictors with the presence of PDDIs. whereas, the individual effect of predictors was also evaluated by applying univariate logistic regression.

Univariate logistic regression revealed a positive, statistically significant association between the presence of PDDIs with the following independent variables: > 6 prescribed drugs, > 3 days stay in the critical care unit and diagnosis of myocardial infarction, cerebrovascular accident and acute coronary syndrome.

Multivariate logistic regression analysis revealed that the presence of PDDIs was 2.8 times more likely in patients prescribed > 6 drugs, and 0.5 times more likely in patients of age > 75 years. The results of univariate and multivariate logistic regression are shown in Table 3.

**Table 3:** Factors associated with drug-drug interaction using logistic regression (*n* = 1044)

Variable	Univariate Regression		Multivariate Regression	
	OR (95%CI)	*p*-value	OR (95%CI)	*p*-value
*Prescribed drugs*				
≤ 6	Reference		Reference	
> 6	2.172 (1.590–2.968)	< 0.001	2.870 (2.011–4.095)	< 0.001
*Age*				
≤ 75	Reference	0.106	Reference	
> 75	0.748 (0.526–1.064)		0.588 (0.386–0.897)	< 0.05
*Duration of stay*				
≤ 3	Reference	< 0.001	Reference	
> 3	0.622 (0.472–0.820)		0.868 (0.626–1.204)	0.397
*Gender*				
Male	Reference	0.250	Reference	
Female	0.853 (0.650–1.119)		0.820 (0.595–1.129)	0.224
*Chronic illness*				
Myocardial infarction	0.018 (0.005–0.058)	< 0.001	0.012 (0.003–0.040)	< 0.001
Cerebrovascular accident	0.192 (0.080–0.463)	< 0.001	0.147 (0.059–0.367)	< 0.001
Acute coronary syndrome	0.043 (0.011–0.162)	< 0.001	0.037 (0.009–0.142)	< 0.001
Heart failure	0.384 (0.157–0.936)	< 0.05	0.265 (0.105–0.673)	< 0.05

*CI* Confidence interval

A total of 225 drug combinations were involved in all the PDDIs, of which 70.2% attributed to 16 pairs. The common interacting pairs along with their potential outcomes and management are shown in Table 4.

**Table 4:** Characteristics of common drug-drug interactions

Drug combination	Frequency	Severity	Documentation	Potential outcome	Mechanism	Management
Aspirin & clopidogrel	420	Major	Fair	Increased risk of bleeding	Synergism	Monitor blood count
Clopidogrel & Enoxaparin	277	Major	Fair	Increased risk of bleeding	Synergism	Monitor blood count
Aspirin & Ramipril	186	Moderate	Fair	Decreased Ramipril effectiveness	Inhibition of prostaglandin synthesis	Replace Ramipril with suitable drug
Aspirin & Diuretics	239	Major	Good	Reduced diuretic effectiveness and risk of nephrotoxicity	Decreased renal prostaglandin synthesis	Monitor for any changes
Aspirin & Betablockers	251	Moderate	Good	Antihypertensive effect of beta blockers may be reduced	Decreased renal prostaglandin synthesis	Monitor for any changes
Aspirin & Nitroglycerin	156	Moderate	Good	Increased nitroglycerin plasma concentration	Synergism	Monitor for any changes
Clopidogrel & Atorvastatin	97	Moderate	Excellent	Decreased formation of clopidogrel active metabolite	Competition with CYP3A4	Replace with rosuvastatin or pravastatin
Ramipril & Furosemide	69	Moderate	Good	Postural hypotension	Vasodilation	Monitor for any changes
Aspirin & Ranitidine	59	Minor	Excellent	Decreased plasma levels of aspirin	Reduced absorption of aspirin	Monitor for any changes
Clopidogrel & Omeprazole	37	Major	Excellent	Decreased plasma concentration of clopidogrel	Inhibition of CYP2C19 mediated clopidogrel metabolism.	Use pantoprazole instead of omeprazole
Dexamethasone & Nimodipine	33	Major	Fair	Decreased plasma concentration of nimodipine	Induction of CYP3A4 mediated metabolism of nimodipine	Replace with suitable drug

One-way ANOVA revealed that there was a significant difference (*p* < 0.001) in the means of PDDIs among all the hospitals. Post hoc test showed that there was a significant difference (*p* < 0.05) in the means of PDDIs between NWGH and the rest of the hospitals. Moreover, there was a significant difference (*p* < 0.001) in the means of PDDIs among all the units. Post hoc test showed that there was a significant difference (*p* < 0.05) in the means of PDDIs between CCU and the rest of the units and between SU and the rest of the units.

## Discussion

Previously neglected, the prevalence of PDDIs and their potential outcomes in geriatrics were studied for the first time in Pakistan. The present study revealed a high prevalence of PDDIs in the elderly population. This coincides with the results of other studies conducted in geriatrics and patients admitted to other critical care units [3, 11]. The prevalence of PDDIs was higher in NWGH, which is a private hospital as compared to the other three hospitals. This variation in prevalence may be due to the higher number of prescribed drugs per patient in NWGH. PDDIs is of great concern in elderly population, due to the physiological changes that occur with aging, which may lead to an increased risk of adverse effects due to drug-drug interactions.

The risk of cardiovascular diseases increases with age [12], and the current study also reported cardiovascular disorders to be the most prevalent in the study population. Cardiovascular disorders were also significantly associated with the presence of PDDIs. This emphasizes for greater care when dealing with geriatrics with a cardiovascular disorder.

PDDIs of major and moderate severity were prevalent. A limited number of other studies have observed the categories of PDDIs in elderly. Studies conducted in elderly population at tertiary hospitals reported PDDIs of moderate severity to be the most prevalent [3, 13]. Another study conducted in geriatrics in outpatient settings reported that most of the patients had PDDIs of major severity [14]. Similarly, studies conducted in ICU’s also reported moderate and major severity PDDIs to be among the most prevalent [15, 16]. PDDIs of pharmacodynamic nature were prevalent in the present study due to the involvement of cardiovascular drugs, the mechanism of interaction of most of them was synergistic or antagonistic.

Furthermore, a significant relationship was observed between PDDIs with polypharmacy and age. A cross-sectional study conducted in Brazil also reported a similar significant association between PDDIs with polypharmacy and age [17]. Another research observed a strong association between polypharmacy and negative clinical consequences in elderly population [18]. A Swedish study also reported a similar relationship between drug-drug interactions and increasing number of prescribed drugs [19]. A US study also found a significant association between PDDIs and polypharmacy [20].

Aspirin was involved in most of the prevalent PDDIs. This drug is one of the most common drug used in cardiovascular disorders, however due to its potential for an interaction with other drugs, its use must be continuously monitored for any adverse effects. The frequent use of aspirin and its potential for involvement in PDDIs has also been reported by Tushar et al. in geriatric outpatients [14].

### Limitations

The current study design could not measure the actual adverse clinical outcomes of the PDDIs, for which further studies have to be conducted. Furthermore, these results are specific for geriatrics so must be generalized with caution in pediatric and adult population.

## Conclusion

A higher prevalence of PDDIs in geriatrics poses a great health concern, due to the weak physiological condition of the aging population. Thus, avoidance of PDDIs and managing them appropriately becomes vital. Monitoring systems should be placed in developing countries to monitor not only PDDIs but also other drug related problems to provide quality health care to patients. Moreover, replacing the drugs involved in PDDIs with appropriate drugs having a lesser potential for PDDI can be implemented to further reduce the risk of PDDIs. Education and training regarding this must be provided to the health care professionals.

## Additional file

Additional file 1: Geriatric patient data. This file contains the demographic data and medication profile collected from the treatment charts of the patients. (XLS 3520 kb)

## Abbreviations

CCU: Cardiac care unit
HMC: Hayatabad medical complex
KTH: Khyber teaching hospital
LRH: Lady reading hospital
MICU: Medical intensive care unit
NWGH & RC: Northwest general hospital and research center
PDDIs: Potential drug-drug interactions
SICU: Surgical intensive care unit
SU: Stroke Unit
